# The American Medical Association

**Published:** 1887-07

**Authors:** 


### EDITORIAL.

### The American Medical Association.

At the recent meeting of the American Medical Association the following resolution was offered and remarks made upon the same by Dr. N. S. Davis of Chicago. The resolution was passed by unanimous vote. The purport and outreach of the resolution will be apparent to every reader.

"*Resolved:* That the regular graduates of such dental and oral schools and colleges as require of their students a standard of preliminary or general education and a term of professional study equal to the best class of the medical colleges of this country, and embrace in their curriculum all the fundamental branches of medicine, differing chiefly by substituting practical and clinical instruction in dental and oral medicine and surgery, instead of clinical medicine and surgery, be recognized as members of the regular profession of medicine, and eligible to membership in this Association on the same conditions and subject to the same regulations as other members."

Dr. Davis in introducing the resolution said he wished to explain its object. There are two objects to be had in view, first to relieve a degree of embarrassment that exists between the regular profession as we consider it, and the profession of dentistry. The department of dental and oral surgery is a part of the profession of medicine as much as the department of ophthalmology or otology or any other ology. Our teeth and mouths are a part of our system as much as any other part, and are used more than any other. The embarrassment is this, that in the history of the development of dentistry it originated mostly in mechanical operations. Steadily it has, advanced, and in years gone by—quite a number of years ago our lamented S. D. Gross made a proposition that an oral and dental section be provided as a section in this Association. It was seconded by Dr. Sayre and myself, and it was organized. The International Medical Congress of 1881 provided a section for dental and oral surgery. The Congress to be held in Washington has done the same thing, and it will be one of the most thorough and best organized sections in the Congress. There is an embarrassment in this respect: It is to know just who and by what line of demarkation those engaged in that department shall be recognized as members of the regular profession. Now it is proposed to make a line and draw it where this Resolution says, that all those who are qualified by general education and a course of study equal to the best medical colleges, a curriculum embracing the entire fundamental principles of medicine with the provision that instead of special instruction in clinical medicine and surgery, instruction may be had in dental and oral surgery, shall be recognized as members of the profession of medicine. It will take away a sort of embarrassment. There is a more far reaching, and more valuable underlying object in this resolution, and that is that to be recognized as a member of the profession, if this resolution is adopted by this body, they must have the education received in schools that require these qualifications, it makes a strong lever to lift up the course of study in the dental schools. Such are my reasons for bringing up the resolution. I will say nothing more on the subject.

The motion was made that the resolution be adopted by the Association and it was carried unanimously.

